# Dietary Glucose Consumption Promotes RALDH Activity in Small Intestinal CD103^+^CD11b^+^ Dendritic Cells

**DOI:** 10.3389/fimmu.2020.01897

**Published:** 2020-08-11

**Authors:** Hyun-Ja Ko, Sung-Wook Hong, Ravi Verma, Jisun Jung, Minji Lee, Nahyun Kim, Daeun Kim, Charles D. Surh, Kwang Soon Kim, Dipayan Rudra, Sin-Hyeog Im

**Affiliations:** ^1^Academy of Immunology and Microbiology, Institute for Basic Science, Pohang-si, South Korea; ^2^Division of Integrative Biosciences and Biotechnology, Department of Life Sciences, Pohang University of Science and Technology, Pohang-si, South Korea; ^3^ImmunoBiome Inc., Pohang-si, South Korea

**Keywords:** retinal dehydrogenase (RALDH), regulatory T cells (Treg), dendritic cells (DCs), LP-DCs, retinoic acid (RA), vitamin A, immune regulation, dietary glucose

## Abstract

Retinal dehydrogenase (RALDH) enzymatic activities catalyze the conversion of vitamin A to its metabolite Retinoic acid (RA) in intestinal dendritic cells (DCs) and promote immunological tolerance. However, precise understanding of the exogenous factors that act as initial trigger of RALDH activity in these cells is still evolving. By using germ-free (GF) mice raised on an antigen free (AF) elemental diet, we find that certain components in diet are critically required to establish optimal RALDH expression and activity, most prominently in small intestinal CD103^+^CD11b^+^ DCs (siLP-DCs) right from the beginning of their lives. Surprisingly, systematic screens using modified diets devoid of individual dietary components indicate that proteins, starch and minerals are dispensable for this activity. On the other hand, in depth comparison between subtle differences in dietary composition among different dietary regimes reveal that adequate glucose concentration in diet is a critical determinant for establishing RALDH activity specifically in siLP-DCs. Consequently, pre-treatment of siLP-DCs, and not mesenteric lymph node derived MLNDCs with glucose, results in significant enhancement in the *in vitro* generation of induced Regulatory T (iTreg) cells. Our findings reveal previously underappreciated role of dietary glucose concentration in establishing regulatory properties in intestinal DCs, thereby extending a potential therapeutic module against intestinal inflammation.

## Introduction

Intestinal dendritic cells (DCs) are critical for the initiation and regulation of innate and adaptive immunity by delivering self or foreign antigens to T cells (1–3). The intestine is spontaneously exposed to innumerable antigens comprising of intestinal microbes (4, 5) as well as dietary components (6, 7). To maintain immune homeostasis, intestinal DCs regulate the balance between the tolerogenic immune response by inducing CD4^+^Foxp3^+^ regulatory T cells (Treg cells) (8–10) and the protective immune responses by inducing effector T cells (11, 12). Dysregulation of this balance by harmful pathogens or dietary intake results in inflammatory disorders (13, 14), such as inflammatory bowel disease (IBD) (15, 16), celiac disease (17) and food allergy (18).

Intestinal DCs are located in the Peyer's Patches (PPs), mesenteric lymph nodes (MLNs) and lamina propria (LP) and comprise cellular subsets that have different origins and functions (1, 2, 19–23). Among these DC subtypes, intestinal CD103^+^ DCs have the unique function that metabolizes vitamin A to retinoic acid (RA) through the activation of aldehyde dehydrogenase 1, member A2 [Aldh1a2, also called retinaldehyde dehydrogenase (RALDH2)] enzyme (24, 25). The RA produced by intestinal DCs play an important role in orchestrating immune responses; imprinting gut-homing specificity on T cells, B cells and innate lymphoid cells (ILCs), inducing IgA-producing B cells, promoting TGF-β-dependent differentiation of induced Treg cells, suppressing the differentiation of Th17 cells, enhancing IL-22 production by γδ T cells and ILCs, as well as inducing effector functions in T cells (26–39).

While vitamin A derived from dietary intake can induce RALDH enzymatic activity, the RA produced from intestinal epithelial cells (IECs) by RALDH1 and stroma cells in LP and MLN by RALDH2, in a trans activating mechanism is also capable of inducing RALDH expression in intestinal DCs (40–44). Furthermore, recent data suggest that RA is also involved in the development of a gut homing precursor for intestinal DCs in the bone marrow as well as is required for their transcriptional programming and maturation (45, 46). Several endogenous factors that regulate RALDH expression in LP-DCs are also reported. Cytokines such as IL-4 and granulocyte macrophage colony-stimulating factor (GM-CSF) induce or enhance the expression of RALDH enzymes in LP-DCs, while prostaglandin E2 (PGE2) negatively regulates RALDH activity through the induction of inducible cyclic AMP early repressor (ICER) (24, 47–49). Despite these findings, whether additional components in diet can induce RALDH activity in the intestine and promote immune tolerance, remains unknown. In this study, we uncover a hitherto unknown role of dietary glucose in shaping up intestinal immunological tolerance by facilitating RALDH expression specifically in intestinal LP-DCs.

## Results

### Mice Administered Antigen Free Diet Have Defects in Development and RALDH Activity in CD103^+^CD11b^+^ siLP-DCs

**Figure 1:**
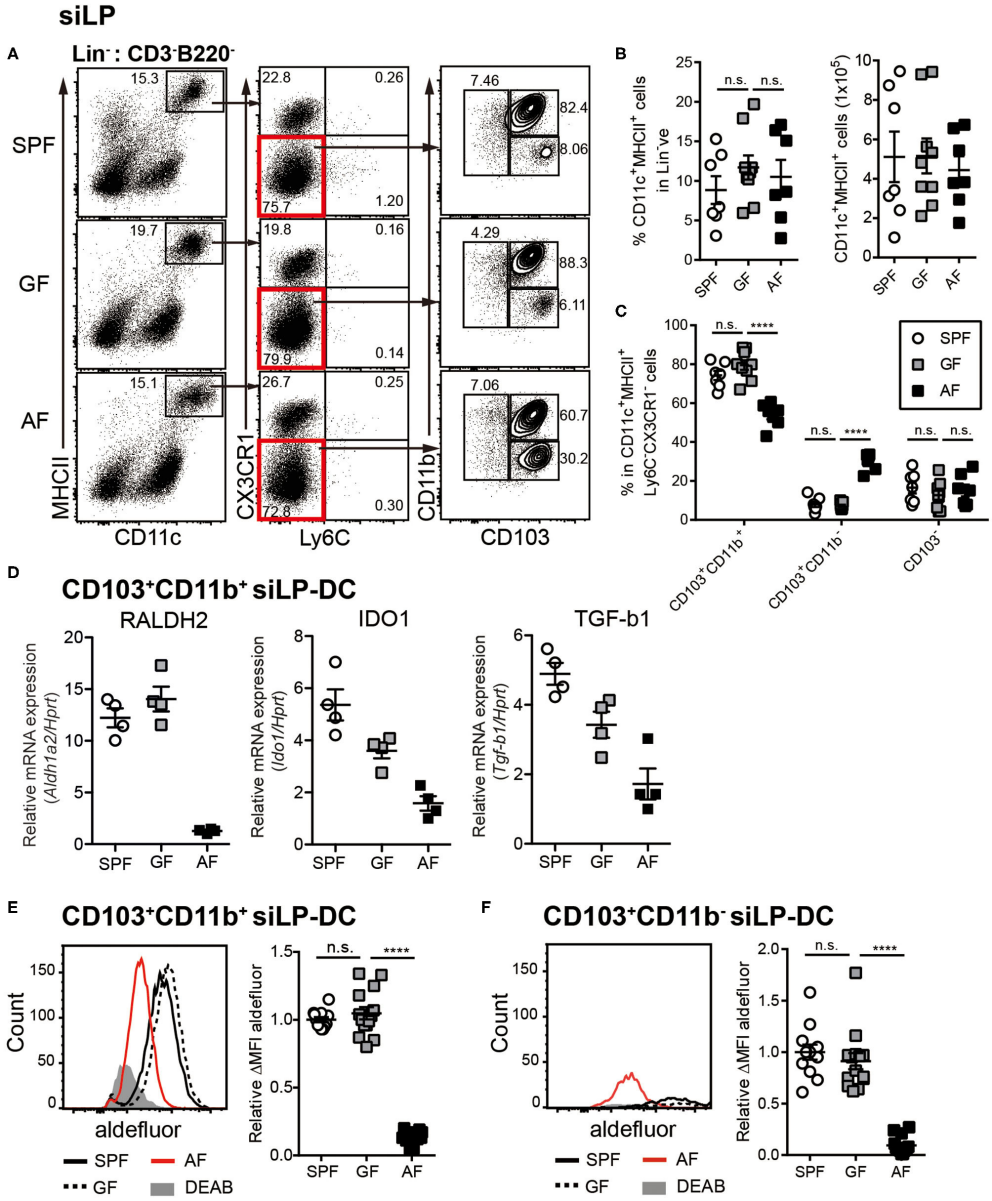
Dietary intake affects differentiation and RALDH activity in CD103^+^ siLP-DCs. Cell suspensions were prepared from siLP harvested from age-matched adult (6~12-week-old) SPF, GF and AF mice and phenotypic and RALDH activity analyses of siLP-DCs were carried out. **(A)** Representative fluorescence-activated cell sorting (FACS) plots of siLP-DC subpopulations gated on Lin^−^ (CD3^−^B220^−^) cells. **(B)** Statistical quantification of percentage (left) and total numbers (right) of CD11c^+^MHC-II^+^ cells in mice from indicated experimental groups. **(C)** Graph displays percentage of siLP-DC subpopulations in CD11c^+^MHC-II^+^ cells. **(B,C)** Data are combined from four independent experiments. **(D)** Real-time analyses of mRNA expression of indicated gene products normalized against *Hprt* mRNA levels. **(E,F)** Representative FACS plots (left) and quantification (right) of relative mean fluorescence intensity (MFI) of aldefluor in CD103^+^CD11b^+^ **(E)** and CD103^+^CD11b^−^ **(F)** siLP-DC subpopulations from indicated groups. DEAB is a RALDH inhibitor. ΔMFI is calculated by subtracting background (DEAB) MFI from aldefluor MFI. Relative ΔMFI indicates ratio of ΔMFI in experimental samples vs. control. Data are combined from six independent experiments. MEAN ± SEM are indicated. Statistical significance was determined by one-way ANOVA **(B,E,F)** and two-way ANOVA **(C)** with Turkey's multiple comparison tests. ****p < 0.0001, *n.s*., not statistically significant.

To investigate the influence of commensal microbiota and food components on intestinal immunity, we utilized the previously established “Antigen free (AF)” mice model, where germ-free (GF) mice are raised on well-defined elemental diet [termed “Antigen free diet” (AFD)] devoid of macromolecules such as proteins and starches (50). When DCs in small intestine were assessed, we observed comparable frequencies of CD11c^+^MHC-II^+^ siLP-DCs in specific pathogen free (SPF), GF and AF mice (Figures 1A,B). However, in-depth analyses revealed alteration in the frequencies of tolerogenic DC subtypes. The frequency of CD103^+^CD11b^+^ siLP-DCs, a subset known to be a major tolerogenic DC population, was slightly, but significantly lower in AF, when compared to SPF and GF mice (Figure 1C). A compensatory increase on the other hand was observed in the CD103^+^CD11b^−^ siLP-DC compartment. More interestingly, while the expression of the characteristic DC surface markers largely remained comparable in all three groups (Figure S1A), the expression of all three representative genes tested, namely *Aldh1a2*, Indoleamine-pyrrole 2,3-dioxygenase 1 (*Ido1*) and Transforming growth factor beta 1 (*Tgfb1*) that are functionally implicated in tolerogenic phenotype of CD103^+^CD11b^+^ DCs, were dramatically reduced in AF mice (Figure 1D). Interestingly, the expression of *Aldh1a2* was found to be specifically reduced in mice raised under AFD, a phenomenon that was not observed in GF mice (Figure 1D, left panel). On the other hand, the absence of gut microbiota appeared to partially influence the expression of *Ido1* and *Tgfb1*, which was further enhanced by AFD (Figure 1D, middle and right panel). These results indicated that certain dietary components, otherwise absent or under-represented in AFD have most specific and the largest influence on the expression of *Aldh1a2*. For this study, we therefore focused on the influence of normal diet on RALDH activity in siLP-DCs.

RALDH is an enzyme that irreversibly metabolizes vitamin A to RA, which in turn acts as a key modulator of mucosal immune responses (38, 51–53). To determine whether in concert to its reduced expression, the function of RALDH in LP-DCs from AF mice was also negatively affected, we next examined RALDH enzyme activity in LP-DCs from SPF, GF and AF mice using the ALDEFLUOR assay. In this assay, which has been previously employed in the context of CD103^+^ LP-DCs and MLN-DCs (43, 44), the RALDH enzyme activity is measured in individual cells by flow cytometry with a fluorescent substrate based assay system (54, 55). In agreement with the results obtained by real-time PCR analysis, CD103^+^ siLP-DCs (both CD11b^+^ and CD11b^−^ subsets) from AF mice displayed significantly lower enzyme activity when compared with SPF and GF mice (Figures 1E,F, Figure S1B). Of note, the characteristic frequencies of the aforementioned siLP-DC subtypes in SPF, GF and AF mice remained unaltered even after performing this assay, suggesting that this enzyme assay did not interfere with the phenotype of siLP-DCs (Figure S1C).

**Figure 2:**
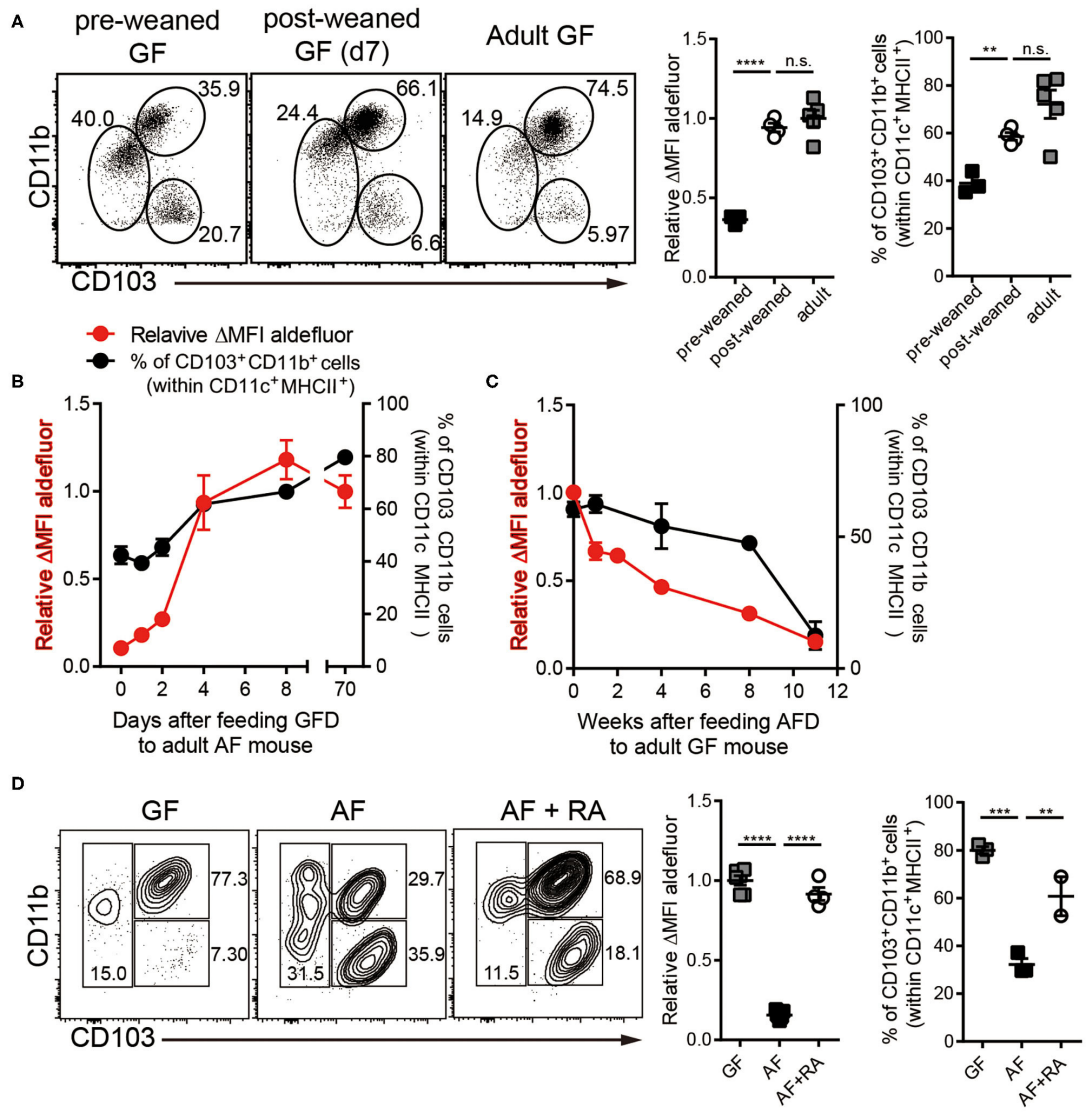
Dietary components in normal chow readily trigger and maintain RALDH activity in siLP-DCs in mice after weaning. Cell suspensions prepared from siLP were subjected to ALDEFLUOR assays and RALDH activity in CD103^+^CD11b^+^ siLP-DCs and percentage of siLP-DC subpopulations in CD11c^+^MHC-II^+^ cells were analyzed by flow cytometry. **(A)** GF mice (3-weeks old) before weaning (pre-weaned), GF mice weaned onto NCD for 7 days and adult GF mice were analyzed. **(B)** RALDH activity and frequencies of siLP-DC subpopulations in CD11c^+^MHC-II^+^ cells in adult AF mice (8~12-week-old) after feeding NCD for 0, 1, 2, 4, 8, and 70+ days. **(C)** RALDH activity and frequencies of siLP-DC subpopulations in CD11c^+^MHC-II^+^ cells in adult GF mice (8~12-week-old) after feeding AFD for 0, 1, 2, 4, 8, and 11+ weeks. **(D)** RALDH activity and frequencies of siLP-DC subpopulations in CD11c^+^MHC-II^+^ cells in adult GF, AF, or AF mice that were administered intra peritoneal injection of all-trans RA (500 μg per mouse in soybean oil) every other day for 7 days. Data are combined from two to three independent experiments. MEAN ± SEM are indicated. **(B,C)** Statistical significance was determined by one-way ANOVA with Turkey's multiple comparison test. **p < 0.01, ***p < 0.001, ****p < 0.0001, *n.s*., not statistically significant.

In order to further understand the role of dietary components on RALDH activity, we next analyzed siLP-DC RALDH activity in mice at different stages of their lives, after subjecting them to specific dietary conditions. We observed that RALDH activity in pre-weaned GF mice (2-3 weeks of age) was significantly lower than in adult GF mice, and comparable to AF mice. This was dramatically restored to the level equivalent to adult GF mice within a week after weaning (Figure 2A). Furthermore, when mice raised in AF condition were fed with Normal Chow Diet (NCD), RALDH activity in CD103^+^CD11b^+^ siLP-DCs was promptly recovered within a week (Figure 2B). Mirroring this, an opposite phenomenon was observed when NCD in GF mice was replaced with AFD (Figure 2C). These results suggested that dietary component(s) in NCD, absent in AFD, is required as the initial trigger for RALDH gene expression after mice are weaned, thereby promoting enzyme activity as well as homeostasis of CD103^+^CD11b^+^ siLP-DCs.

The above results also implied that supplementing AF mice with RA, the final product of the enzymatic reaction and a known feedback inducer of RALDH activity (40), would be sufficient in driving RALDH activity in these mice. Indeed, when adult AF mice were supplemented in their diet with RA, it resulted in complete recovery of RALDH activity (Figure 2D). Interestingly in all the cases, changes in RALDH activity also correlated with the frequencies of CD103^+^CD11b^+^ siLP-DCs, suggesting its role in differentiation as well as function of these cells. Of note, while the above results were obtained in a GF setting, the basic findings from these experiments could also be recapitulated in mice raised in SPF conditions, thereby confirming that mice with normal repertoire of gut flora are equally affected by dietary components with respect to RALDH activity in siLP-DCs (Figures S2A–C).

### RALDH Activities in Different Intestinal RA-Producing Cells Are Differentially Affected by Diet

We next sorted to understand whether the influence of diet on RALDH activity is an LP-DC specific phenomenon, or whether other RALDH expressing cells are also affected. It is well-established that within the gut associated lymphoid tissues, besides LP-DCs, RA converting enzymes are also expressed in LP associated stroma cells (LP-SCs), small intestine epithelial cells (IECs), as well as MLN-DCs. The RA produced from these cells, in particular IECs, is known as a local source of RA for inducing the RALDH expression in CD103^+^ siLP-DCs (40–44).

**Figure 3:**
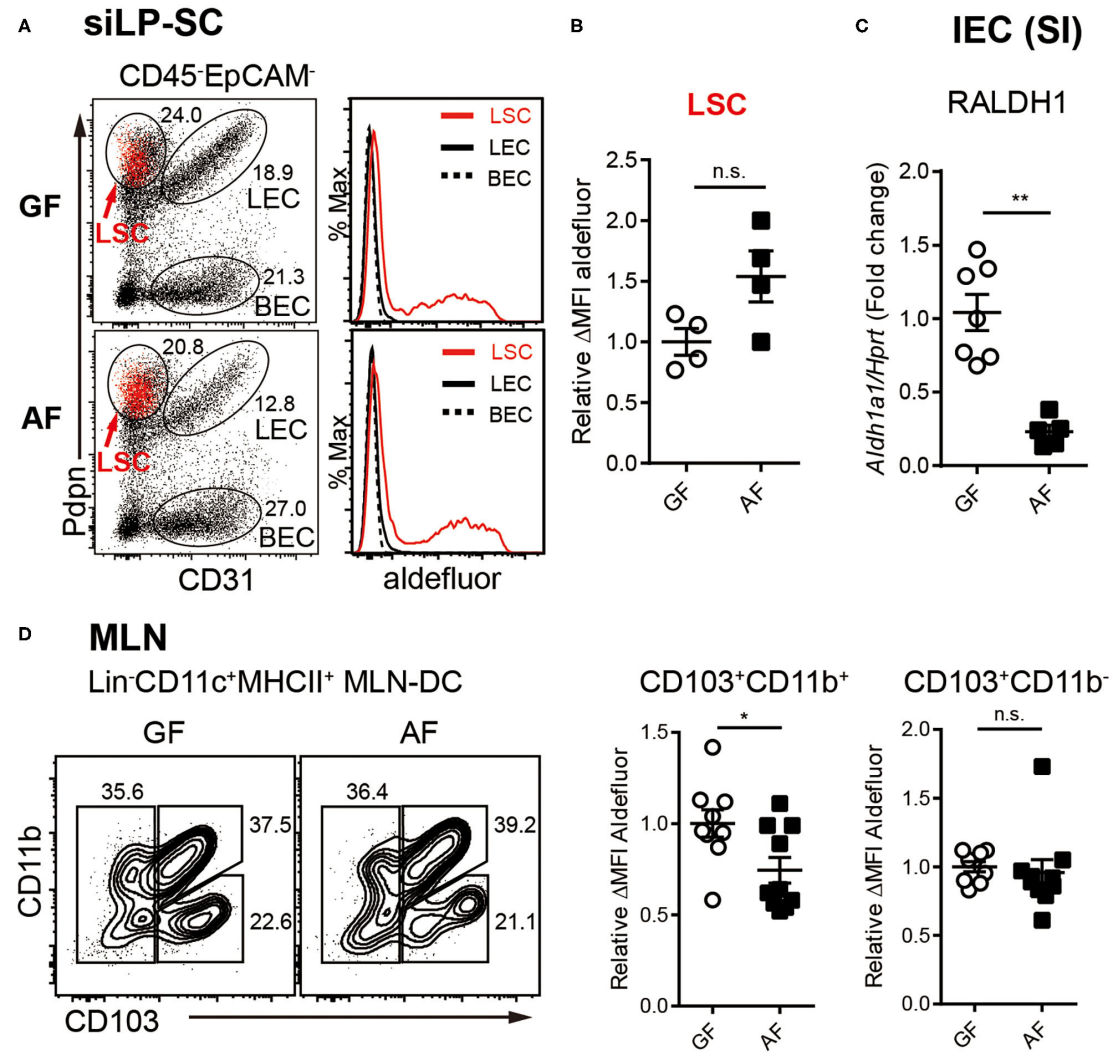
RALDH activity in intestinal RA-producing cells is differentially regulated by diet. **(A–C)** Cell suspensions from MLN and siLP harvested from age-matched adult (6~12-week-old) GF and AF mice were subjected to ALDEFLUOR assays and RALDH activity of LP-SCs and MLN-DCs were analyzed by flow cytometry. **(A)** Representative FACS plot of LP-SC distinguished by CD31 and Pdpn from CD45-EpCAM- cells. Red dots in FACS plot indicate aldefluor positive cells. Histograms depict the MFI of aldefluor in LP-SC subtypes. **(B)** Graph displays the level of RALDH activity in LSCs. Data is combined from two independent experiments. **(C)** IECs were isolated from small intestine (SI) by stripping with EDTA and analyzed for expression of *Aldh1a1* (RALDH1) by real-time PCR, upon normalization to *Hprt* mRNA levels. Fold change indicates ratio target gene in experiment/control. Data are combined from four independent experiments. **(D)** Representative FACS plots of MLN-DC subpopulations distinguished by CD103 and CD11b from CD11c^+^MHC-II^+^Lin^−^ cells and the RALDH activity in CD103^+^CD11b^+^ MLN-DCs and CD103^+^CD11b^−^ MLN-DCs. Data are combined from four independent experiments. MEAN ± SEM are indicated. Statistical significance was determined by two-tailed unpaired t-test. *p < 0.05, **p < 0.01, *n.s*., not statistically significant.

The non-hematopoietic SCs in secondary lymph nodes comprise three different cell types based on the expression of surface markers. Lymphatic stroma cells [LSCs, also called fibroblast reticular cells (FRC)], lymphatic endothelial cells (LECs) and blood endothelial cells (BECs) (56, 57). Among LP-SCs (CD45^−^EpCAM^−^) in small intestine, the LSCs that expressed podoplanin (Pdpn) and are CD31^−^, were found to be capable of activating RALDH enzymes (Figure 3A), as previously reported (43). Interestingly, unlike LP-DCs, the RALDH activity in LP-SCs remained comparable between GF and AF mice (Figure 3B). In contrast, when IECs were analyzed, the expression of *Aldh1a1* (RALDH1), the major gene encoding for RALDH enzyme in these cells, was found to be reduced in AF mice (Figure 3C). Of note, while the IECs are well-established to have RALDH activity (27, 58–61), the baseline of this activity in these cells is known to be significantly lower than LP-DCs (62, 63). Therefore, our attempt to measure RALDH activity in IECs was unsuccessful due to lower sensitivity of the ALDEFLUOR assay. However, albeit comparatively lower RALDH activity on a per cell basis, the cumulative contribution of IECs in RA production, is understandably of larger significance since numerically there are many more IECs than the other cell types in the intestine.

Finally, when MLN-DCs were analyzed, the RALDH activity in particular within the CD103^+^CD11b^+^ DC population in AF mice was found to be significantly, albeit to a lesser extent, lower than that of GF mice (Figure 3D). Taken together these results suggested that dietary components differentially influence RALDH activity in different regulatory DC populations. Whereas, MLN-DCs and IEC are affected, LP-SCs appeared to remain unaffected from dietary contributions. These results also implied that the overall reduction of RA synthesis cumulatively among these cell types eventually contributed to the reduced RALDH activity in the LP-DCs in AF mice.

### Proteins, Starches, and Minerals in Diet Do Not Influence RALDH Activity in CD103^+^CD11b^+^ siLP-DCs

Since for this study we focused on RALDH activity in siLP-DCs, we next wished to define which dietary factors were required to trigger the initial RALDH activity in these cells. While in our initial findings we observed both the subtypes CD103^+^CD11b^+^ and CD103^+^CD11b^−^ siLP-DCs to have reduced RALDH activity in AF mice (Figures 1E,F), the cell recovery of CD103^+^CD11b^−^ siLP-DCs from SPF and GF mice were low and the level of enzyme activity in this cell type showed variability among individual mice (Figures 1C,F). Therefore, henceforth in this study, we focused on the RALDH activity in CD103^+^CD11b^+^ siLP-DCs.

We first confirmed that the reduction in RALDH activity in CD103^+^CD11b^+^ siLP-DCs was not a consequence of low Vitamin A content in AF diet, thereby compromising the precursor for the assayed reaction. Based on information of food compositions from the suppliers and from our pervious report, final consumption of vitamin A per day by GF and AF mice were comparable [Table S1 and (50)]. Nonetheless, there remained a possibility that albeit equal consumption, the absorption of vitamin A into the small intestine may be compromised in AF mice. However, when GF mice were weaned on AF diet supplemented with 10 times more vitamin A in the usual form of “oil mix” or were administrated additional “oil mix” by oral gavage, failed to recover the reduction in RALDH activity in CD103^+^CD11b^+^ siLP-DCs (Figure S3). We thus concluded that mere unavailability of the precursor vitamin A was not a cause of reduced RALDH activity in these cells.

To this end, we modified the compositions of purified diet by removing individual food components (Table S2), with the presumption that taking out or adding back individual components in otherwise well-defined diet may lead us toward identifying the dietary component required to trigger RALDH activity. To obtain relatively accurate results under *in vivo* settings, the modified diets were designed to contain similar amount of vitamin A as in NCD (Tables S1, S2) and the experiments were performed primarily in GF condition in order to eliminate the influence of microbiota. In addition, to avoid any influence from NCD during the pre-weaned period, neonate AF mice were utilized and these mice were weaned onto each modified diet for 3–4 weeks.

**Figure 4:**
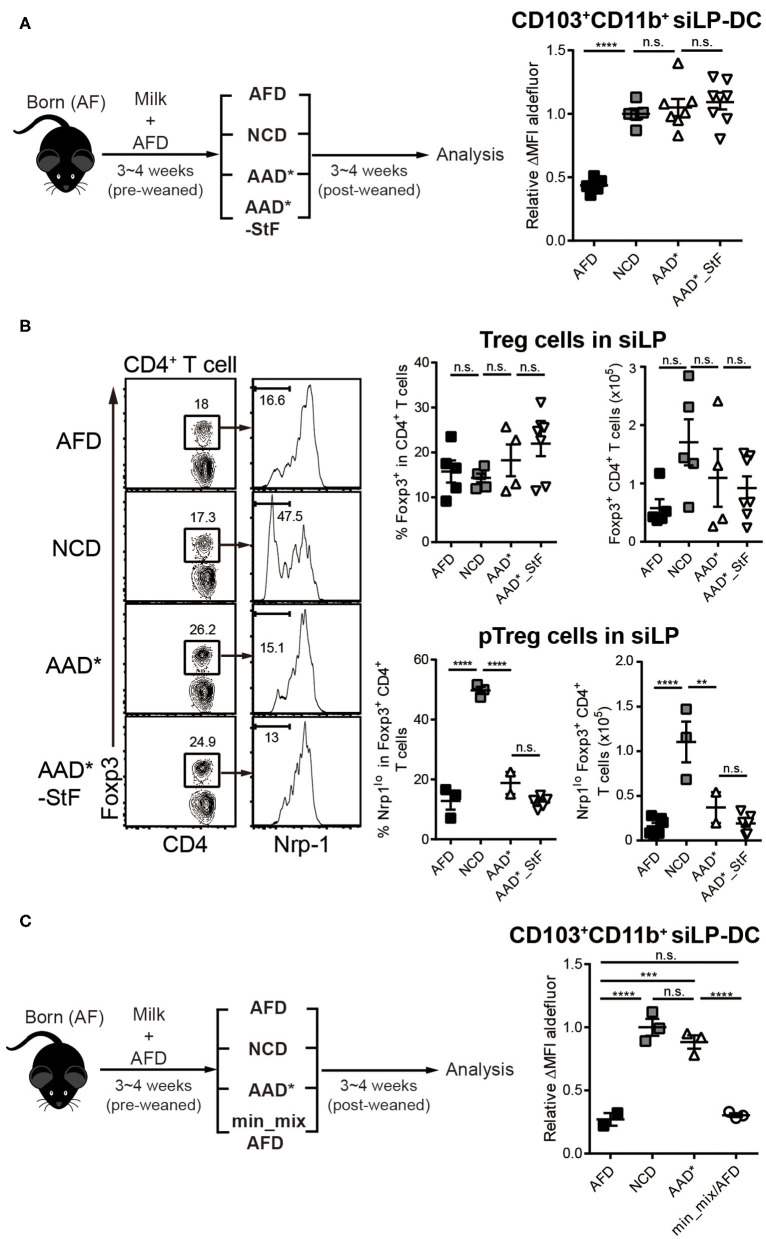
Depletion of macromolecules from purified diet do not alter the RALDH activity in CD103^+^CD11b^+^ siLP-DCs. AF mice (3~4-week-old) were weaned onto specific diets for 3~4 weeks following which the indicated analyses were carried on. **(A)** A cartoon depicting experimental scheme (left panel). AAD* is a sterilized form of Amino acid-defined diet (AAD) that contains three times more vitamin A than AAD, where protein macromolecules are replaced with amino acids. AAD*_StF indicates AAD* from which cornstarch and maltodextrin are removed. The level of RALDH activity in CD103^+^CD11b^+^ siLP-DCs in the indicated groups are presented by relative aldefluor ΔMFI (right panel). **(B)** Representative FACS plots (left panel), frequencies (middle panel) and absolute numbers (right panel) of total CD4^+^Foxp3^+^Treg cell and CD4^+^Foxp3^+^Nrp1^lo^ peripheral Treg (pTreg) cell populations in siLP of mice subjected to the indicated diet regimes. **(C)** Experimental scheme (left panel), and relative aldefluor ΔMFI of siLP-DCs in the indicated experimental groups (right panel). Min_mix AFD indicates AFD mixing with the mineral mix powder (TD.94049). Data are combined from four independent experiments. MEAN ± SEM are indicated. Statistical significance was determined by one-way ANOVA with Turkey's multiple comparison test, **p < 0.01, ***p < 0.001, ****p < 0.0001, *n.s*., not statistically significant.

As a starting point, we took advantage of two commercially available diets with well-defined dietary compositions. In the so-called amino acid defined diet (AAD^*^), like the AFD employed so far, proteins were replaced with amino acids. However, there were several differences between their compositions (Table S2). While AFD is a liquid diet where the fibers are provided as cellulose bedding, AAD^*^ diet are edible solid pellets with cellulose mixed with the food components. Unlike AFD, AAD^*^ diet contained starches in the form of maltodextrin and corn starch, and while the source of sugar in AFD was 22% glucose, that in AAD^*^ was 37.1% sucrose. Furthermore, in terms of mineral composition, there are substantial differences between the groups. The second commercially available diet is AAD^*^_StF, which was largely similar to AAD^*^, but was devoid of starches. Note, the “^*^” in AAD^*^ indicates a sterilizable form of the diet which is otherwise similar to its traditional form (AAD), but with three times more vitamin A to account for presumed losses during sterilization by irradiation. Surprisingly, the mice groups weaned in both AAD^*^ and AAD^*^_StF showed a complete recovery of RALDH activity in CD103^+^CD11b^+^ siLP-DCs, to an extent similar to the control NCD fed group (Figure 4A). These results were independent of microbiota, since the characteristic drop in RALDH activity of CD103^+^CD11b^+^ siLP-DCs in mice raised in SPF conditions could also be recovered by AAD (Figure S2D). Taken together these findings led to two important conclusions. First, starches are not involved. Second, antigens in the form of peptides, derived from proteins are also dispensable as far as RALDH activity in CD103^+^CD11b^+^ siLP-DCs is concerned. To this end, we also considered a possibility that an artifact arising from unaccounted protein contamination in the amino acid defined diets may be responsible for the observed recovery of RALDH activity. We therefore quantified the generation of peripheral Regulatory T (pTreg; also referred to as iTreg when induced *in vitro*) cell population in the siLP of these mice. pTreg cells are a type of Treg cells that are extrathymically generated primarily at mucosal sites, and are distinguished from their thymic tTreg counterparts by the lack of expression of the membrane bound co-receptor neuropilin-1 (Nrp1) (64). Notably, in a previous report we have demonstrated that diet derived proteins are the primary cause for the generation of CD4^+^Foxp3^+^Nrp1^−^ pTreg cells in the small intestine (50), and AF mice display dramatically reduced pTreg population in siLP. Indeed we found that while total Foxp3^+^ Treg populations, comprising of tTreg and pTreg cells, remained comparable, the frequencies and numbers of Foxp3^+^Nrp1^−^ pTreg cells among total Treg population could only be recovered in mice fed with NCD, and not AAD^*^ and AAD^*^_StF (Figure 4B). Therefore, the recovery of RALDH activity in AAD^*^ and AAD^*^_StF groups were not due to any protein contamination in the AAD^*^ diet.

We next wished to exclude the possibility that differences in minor food components such as minerals, between AFD and purified diet (Table S2), was responsible for differences in RALDH activity. For that we prepared AFD by supplementing with mineral mix powder (TD.94049) derived from AAD^*^ (min_mix/AFD). As shown in Figure 4C, min_mix/AFD failed to induce RALDH activity. Taken together, these results concluded that proteins, starches and minerals in diet were not responsible for the induction of RALDH activity in CD103^+^CD11b^+^ siLP-DCs.

### Optimum Glucose Level in Diet Is Required To Induce RALDH Activity in CD103^+^CD11b^+^ siLP-DCs

**Figure 5:**
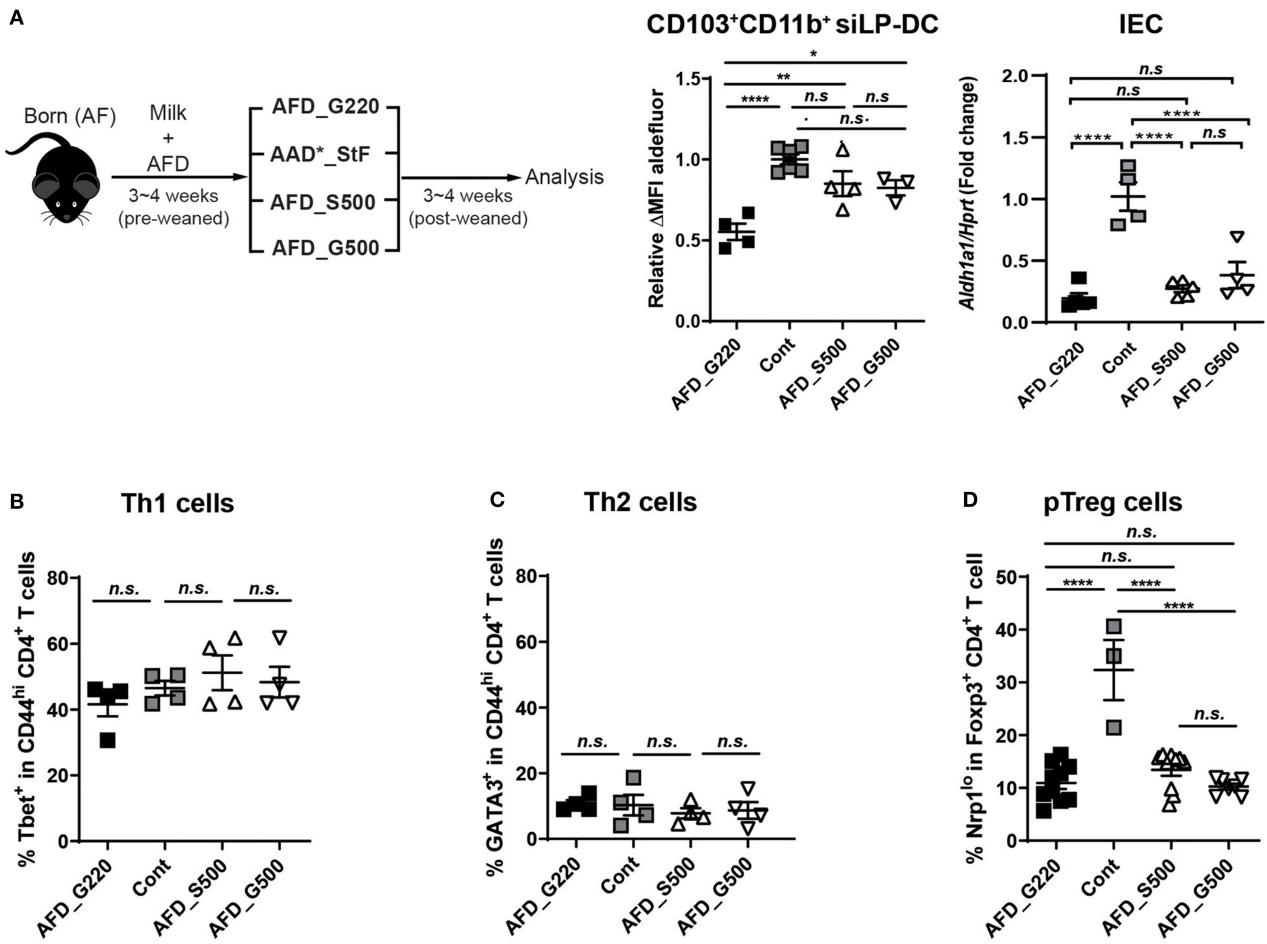
Supplementing glucose in AF diet restores RALDH activity in CD103^+^CD11b^+^ siLP-DCs. **(A)** Cartoon depicting experimental scheme (left panel). Neonate AF mice were weaned onto specific diets for 3~4 weeks. AFD contains 22% of glucose (AFD_G220). AFD_G500 indicates 50% of glucose in AFD and AFD_S500 indicates 50% of sucrose in AFD. AAD*_StF was utilized as a positive control of RALDH activity in LP-DCs. ALDEFLUOR assays were performed on cell suspensions from siLP and RALDH activity of siLP-DC was analyzed by flow cytometry. The level of RALDH activity in CD103^+^CD11b^+^ siLP-DCs is represented as relative ΔMFI of aldefluor (middle panel). *Aldh1a1* expression was also determined in sorted IECs relative to *Hprt* control (right panel). Data are combined from two independent experiments. **(B–D)** Graphs display the percentage of Tbet^+^ in CD4^+^ T cells (Th1) **(B)**, GATA3^+^ in CD4^+^ T cells (Th2) **(C)**, and Nrp1^lo^ populations among in CD4^+^Foxp3^+^ Treg cells (pTreg) **(D)**. Data are combined from at least two independent experiments. MEAN ± SEM are indicated. Statistical significance was determined by one-way ANOVA with Turkey's multiple comparison test, *p < 0.05, **p < 0.01, ****p < 0.0001, *n.s*., not statistically significant.

If the macromolecules and micromolecules in diet were not involved, lack of which factors in AFD compromise RALDH activity in CD103^+^CD11b^+^ siLP-DCs? To investigate further, we compared the food compositions between AAD^*^_StF and AFD (Table S2), which were the closest among the four types of diets tested. In case of AAD^*^_StF, this diet does not contain proteins and starches as in AFD, but does contain unknown dietary factor(s) responsible for the induction of RALDH activity in siLP-DCs. There are few differences between the composition of these two diets; (1) the diet forms (pellet vs. liquid), (2) the carbohydrate sources (sucrose vs. glucose), (3) the amount of carbohydrate (sucrose ~50% vs. glucose ~22%). Based on this observation, as well as in order to minimize the differences in diet forms, we first generated a liquid form of AFD with 50% of sucrose (AFD_S500) or 50% glucose (AFD_G500). Germ free diet (GFD) or AAD^*^_StF were used as positive controls. AFD with usual 22% glucose, designated as AFD_G220 here, was used as negative control. The neonate AF mice were weaned onto each diet for 3–4 weeks and were analyzed for RALDH activity in CD103^+^CD11b^+^ siLP-DCs (Figure 5A, left panel). Indeed, AFD supplemented with 50% sucrose resulted in significantly enhanced RALDH activity in these cells. More interestingly, we observed a similar increase in RALDH activity in CD103^+^CD11b^+^ siLP-DCs even when the source of carbohydrate was changed to 50% glucose instead of sucrose (Figure 5A, middle panel). These effects were found to be specific for CD103^+^CD11b^+^ siLP-DCs, since RALDH1 expression in IECs remained unaltered upon carbohydrate supplementation (Figure 5A, right panel). These results suggested that regardless of the source of carbohydrate, its optimum concentration is important, and glucose being a monosaccharide unit of carbohydrate is sufficient for the initial triggering of RALDH activity in siLP-DCs.

In order to further understand whether the positive effect of dietary carbohydrate supplementation is specific for RALDH activity, or if it can also affect differentiation of other immune cells, we determined the frequencies of Th1, Th2, and pTreg cells in these mice. Supplementation of AFD with additional sucrose or glucose did not affect Tbet^+^ Th1 or Gata3^+^ Th2 cells (Figures 5B,C); neither recovered the characteristically reduced pTreg population in siLP (Figure 5D).

**Figure 6:**
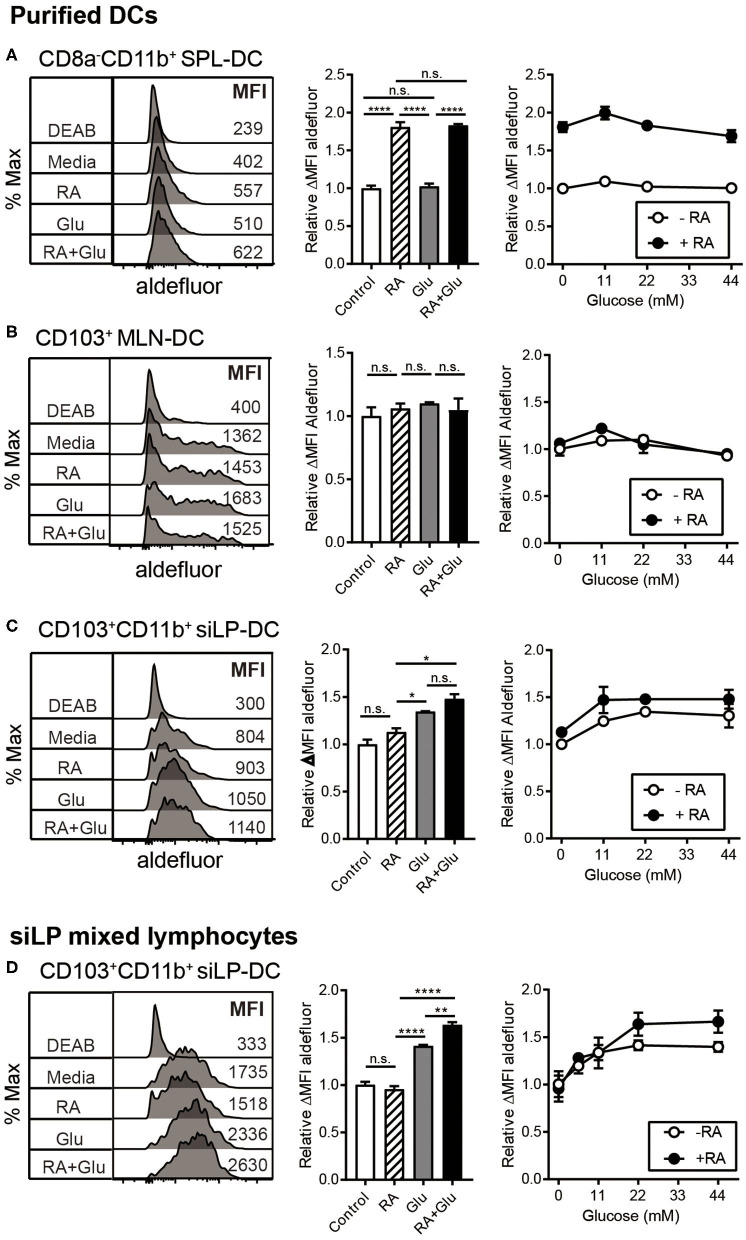
Glucose induces RALDH activity specifically in siLP-DCs. MLN-DCs and Spleen DCs (SPL-DC) from adult SPF mice, or siLP-DCs from 2-weeks old neonatal SPF mice were either purified **(A–C)**, or total single cell suspensions were isolated from adult SPF mice SI **(D)**. Cells were cultured for 20 h in glucose-free media with or without treatments, following which ALDEFLUOR assay was performed. Fluorescence intensities of aldefluor in CD8^−^CD11b^+^ SPL-DCs **(A)**, CD103^+^ MLN-DCs **(B)** and CD103^+^CD11b^+^ siLP-DCs **(C,D)**, were analyzed by flow cytometry. RALDH activity depicted in overlaid histograms and bar graphs are from the cells cultured with glucose (22 mM) or RA (1 nM) or both. The line graphs indicate RALDH activity from the cells cultured with different concentrations of glucose (0, 11, 22, and 44 mM) in the presence or absence of RA (1 nM). Data shown is representative of at least three independent experiments. MEAN ± SEM are indicated. Statistical significance was determined by one-way ANOVA with Turkey's multiple comparison test, *p < 0.05, **p < 0.01, ****p < 0.0001, n.s., not statistically significant.

We next directly investigated the effect of glucose on RALDH activity by employing *in vitro* culture conditions. SPL-DCs, MLN-DCs, and CD11c^+^ siLP-DCs were magnetically purified and cultured either without glucose (in commercially available glucose-free media), without glucose but in the presence of RA, with glucose, or in the presence of glucose and RA for 20 h, and then measured for RALDH activity. In terms of base-line RALDH activity, as expected, SPL-DCs displayed the least, which was significantly increased in the presence of RA. Glucose, on the other hand, had minimal effect (Figure 6A). Although MLN-DCs had the highest base-line RALDH activity among the three groups tested, neither RA nor glucose had an impact (Figure 6B). In contrast, siLP-DCs derived from 2-weeks old neonatal SPF mice which had low basal RALDH activity at steady state, was significantly increased in the presence of glucose alone. While RA itself induced slight increase of this enzyme activity, the highest activity was observed when both RA and glucose was present (Figure 6C). Moreover, this RALDH activity promoting effect of glucose was also observed when siLP mixed lymphocytes derived from 8-weeks old adult mice were cultured with glucose for 20 h. Under the culture conditions tested, RA by itself was found to have minimal effect on the already high RALDH activity, whereas the addition of glucose in the media was able to further boost this activity (Figure 6D).

**Figure 7:**
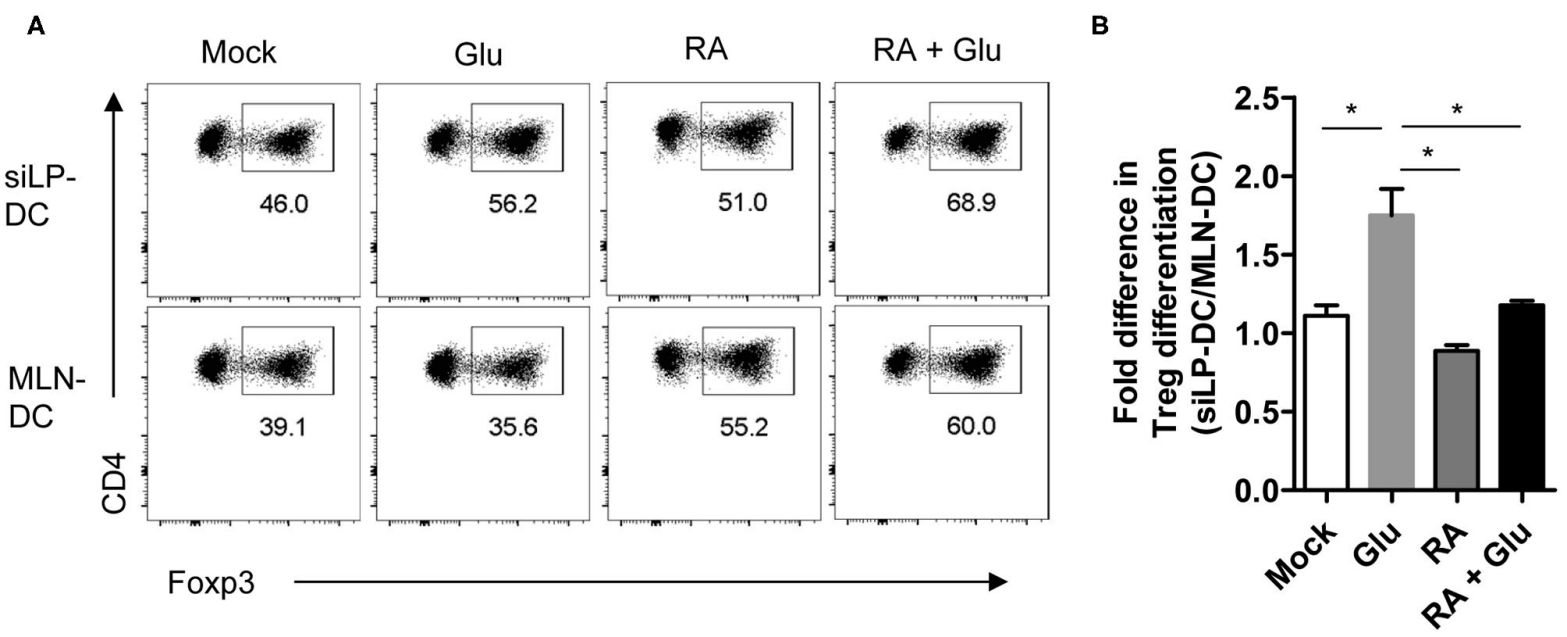
Pretreatment of glucose in siLP-DCs, but not in MLN-DC, significantly induce iTreg cells *in vitro*. siLP and MLN CD11c^+^ DCs were pre-cultured in glucose-free media or media supplemented with 22 mM glucose (Glu), 1nM RA, or with both glucose and RA. After 14 h the DCs were washed and co-cultured with naïve CD4^+^ T cells under suboptimal Treg inducing condition. After 72 h Foxp3^+^ cells was analyzed by FACS. **(A)** Representative FACS plots. **(B)** Bar graphs representing fold differences in Treg induction mediated by siLP-DC vs. MLN-DC. *P < 0.05. Data are representative of two independent experiments with similar results.

Finally, in order to ascertain functional relevance of these findings, we sorted to determine whether supplementation of glucose, presumably through the generation of RA, can facilitate the generation of iTreg cells. For this, we performed an *in vitro* iTreg induction assay with magnetically purified CD11c^hi^ DCs (with “high” CD11c expression) that were pre-treated with glucose and incubated with naïve T cells under suboptimal iTreg inducing condition. We observed significant increase in iTreg induction when siLP-DCs were pre-treated with glucose, compared to mock. This effect of glucose pre-treatment was specific for siLP-DCs, and was not observed when MLN-DCs were used in the assay (Figures 7A,B and Figure S4). These results, in accordance to the results presented in Figure 6B, suggested that the siLP DCs are particularly more susceptible to glucose treatment, and thereby, presumably through enhanced RALDH activity, acquire superior iTreg cell induction capacity compared to MLN DCs. It is to be noted however, that while the purified CD11c^hi^MHCII^+^ DCs used in this assay are present in similar frequencies, there are some site specific differences with regard to the expression of MHCII and CD11c in MLN and siLP. Compared to siLP, MLN has significantly higher proportion of CD11c^−^MHCII^+^ and CD11c^int^MCHII^−^ (with “intermediate” CD11c expression) populations (Figure S4, left panels). Furthermore, the expression of MHCII in purified MLN DCs is lower than that of siLP DCs (Figure S4, right panels). These observations raised the formal possibility that enhanced MHCII expression in siLP, rather than glucose mediated enhanced RALDH activity may be responsible for increased iTreg conversion. However, pre-treatment with RA, either alone or in the presence of glucose, resulted in equally efficient iTreg induction irrespective of the source of the DCs, suggesting that the benefit of glucose pre-treatment is indeed primarily due to enhanced RA production (Figures 7A,B). Lastly, when iTreg induction was carried out in vitro in a DC independent manner, supplementation of the media with excess glucose had little effect, thereby further substantiating the role of DC derived RALDH in this process (Figure S5).

## Discussion

There is accumulating evidence suggesting that vitamin A and its metabolites play a pivotal role in maintaining various biological processes (38, 52, 65–67). Dietary supplementation of RA in the context of cutaneous T cell lymphoma and acute promyelocytic leukemia, have been shown to have beneficial outcome (68–71). Furthermore, many studies highlight anti-inflammatory activities of RA at mucosal sites and tissues, such as intestinal mucosa, airways, lung, central nervous system and skin (72–82). In addition to the effect of RA on cancer and inflammatory diseases, vitamin A or its metabolites play an important role to suppress diet-induced obesity and insulin resistance (83–85). Therefore, a better understanding of the cellular and molecular parameters responsible for vitamin A metabolism is of great biological relevance. In this study by identifying the role of diet, and the importance of glucose consumption for establishing RALDH activity early in life in small intestine DCs, we make substantial contribution to our knowledge related to the role of nutritional components in establishing immunological tolerance at mucosal sites.

By feeding AF diet to mice raised under germ free condition we found that small intestine CD103^+^CD11b^+^ LP-DCs require a dietary component as an initial trigger for RALDH activity. Since vitamin A in diet is known to be essential to activate RALDH and generate RA in LP-DCs, LP-SCs, MLN-DCs, MLN-SCs, and IECs (40, 43, 44), one explanation of this observation could be the possibility that at steady state, AF mice consume less vitamin A compared to GF mice raised on NCD. However, our further experiments in conjunction with a previous report (50) strongly indicate that the availability of vitamin A and the way it is fed does not account for the low RALDH activity in AF mice. Even supplementing large excess of vitamin A to AFD failed to restore the reduction in RALDH activity in neonatal GF mice weaned on AFD compared to NCD, suggesting that a possible inferior intestinal absorption rate of vitamin A in AF mice is unlikely a cause as well.

In order to define the responsible factor in diet, we employed diet regimes with well-defined compositions. First, by weaning neonate AF mice onto amino acid defined diet in which only the proteins were replaced by defined concentration of amino acids we found that protein macromolecules are dispensable. This was a surprising finding and was not due to any unaccounted protein contamination in the AAD^*^ diet, since the same diet failed to induce small intestinal pTreg cells, a phenomenon known to be dependent on peptide based antigen presentation. Furthermore, subsequent experiments using defined diet regimes eliminated starch and minerals as well. There are distinct differences between the purified protein and starch free diet AAD^*^_StF and the AFD. First, the diet forms (pellet vs. liquid), second the carbohydrate sources (sucrose vs. glucose) and third the amount of carbohydrate (sucrose 50 vs. glucose 22%). Since the forms of diet as well as the refinement status of NCD can influence experimental outcome (86), we utilized different types of diets (unrefined NCD, purified diets and liquid form of diet) and compared between these groups. In order to address the first issue, we generated a liquid form of diet containing 50% of sucrose (AFD_S500) and compared with the pellet form of AAD^*^_StF containing around 50% of sucrose. Irrespective of the diet types, both groups showed a similar level of the RALDH activity in LP-DCs and it was significantly higher than AFD that contained 22% of glucose. Sucrose is digested to fructose and glucose by the enzyme sucrase that is secreted from the brush border of the small intestine. Furthermore, a recent study reported that at a steady state, most dietary fructose is metabolized and cleared by the small intestine epithelial cells (87). It therefore appeared possible that the metabolites from dietary fructose may have a role in RA metabolism. However, when liquid diet containing 50% sucrose (AFD_S500) vs. 50% glucose (AFD_G500) were compared, we observed comparable RALDH induction; thereby indicating that appropriate concentration of even a monosaccharide unit of dietary glucose is sufficient to initiate RALDH activity in LPDCs. Of note, while in these experiments the relative RALDH activities were calculated on a per cell basis, the variations in total cells obtained from intestinal lamina propria precluded us from reliably calculating the absolute numbers of RALDH expressing intestinal DCs in mice fed with the different dietary regimes.

Although the above experiments were performed primarily on neonatal AF mice in germ free conditions, several lines of evidence strongly suggested that these changes in RALDH activity in response to dietary glucose is also apparent in SPF mice comprised of normal gut microflora. First, while pre-weaned SPF mice, like GF mice, displayed low RALDH activity in siLP-DCs, its activity was increased when they were fed with NCD after weaning. Second, and converse to this observation, when adult SPF mice were fed with AFD (containing 22% glucose) or pre-weaned SPF mice were weaned onto AFD, they displayed significantly lower RALDH activity in siLP-DCs. Third, this reduction in RALDH activity however was not observed when SPF mice were weaned onto AAD, which has increased carbohydrate content compared to AFD. Taken together these results strongly suggest that the glucose dependent changes in RALDH activity in siLP-DCs is still apparent in mice with normal microbiome content and underscores the physiological relevance of this study.

The composition of carbohydrate in NCD is approximately 44~63% (SPFD and GFD) and 62~67% in purified diets (AIN-93G and AAD^*^), which are significantly higher than the carbohydrate content in mother's milk (6.9–7.2% in human and 3% in mouse) (88, 89). On the other hand, AFD contains 22% glucose which is 2.5-fold lower when compared to control diets. The reason behind this is the fact that the glycemic index (GI) of glucose (103 ± 3) is much higher than that of sucrose (65 ± 4) and fructose (15 ± 4) (90). Therefore, the percentage composition of glucose constituting AFD was calculated by factoring in its GI as a parameter in order to maintain mice healthy and capable of breeding. Indeed, from our previous report and earlier studies (91–93), mice raised in AFD were healthy and normal in size and weight, and displayed no sign of nutritional deficiency. Despite these apparent normalcy however, in this study we found that additional dietary source of carbohydrate is required to trigger LP-DC RALDH activity in AFD fed mice.

As a functional consequence we found that pre-treatment with glucose results in enhanced iTreg inducing property specifically in siLP-DCs in an *in vitro* assay system. This finding is in concert with our previous finding where, in a OTII CD4^+^ T cell transfer based *in vivo* assay we observed significantly reduced pTreg generation in AF, compared to SPF and GF mice (50). We propose that the primary cause of such reduced pTreg conversion if AF mice despite being fed comparable amount of OVA to that in SPF and GF mice, is the lower amount of dietary carbohydrate content in AFD. Taken together our study uncovers the functional significance of minute refinements in dietary compositions and underscores the fact that subtle differences in diet may have significant influence on immune composition and overall organismal physiology.

The role of glycolysis on DC physiology is well-appreciated (94, 95). A recent study further suggests that the level of glucose consumed is capable of reprograming glycolytic metabolism and mitochondrial oxidative phosphorylation in lipopolysaccharide (LPS)-stimulated DCs and repress pro-inflammatory responses (96). The authors propose that the extent of glucose consumption can control the expression of glucose transporters and glycolytic enzymes by mammalian target of rapamycin complex 1 (mTORC1) / hypoxia-inducible factor 1a (HIF1a) / inducible nitric oxide synthase (iNOS) signaling circuit (97–99). There are several studies dating as far back to the late 1950's that point to a possible connection between glucose consumption and vitamin A metabolism (100). In light of these previous reports, in this study we have successfully established an unequivocal role of dietary glucose consumption and RALDH activity in LP-DCs and therefore intestinal tolerance. Precise molecular nature of this connection, and how it affects specifically the LP-DCs, remain less understood, and will be an active area of our investigation in recent future. In sum, these findings significantly enhance our knowledge on the crosstalk between functional relationship between immune homeostasis and dietary intake and expand our understanding of therapeutic strategies for clinical applications.

## Materials and Methods

### Mice

Specific pathogen-free (SPF) C57BL/6 (B6) mice were purchased from The Jackson Laboratory and maintained in the animal facility of POSTECH Biotech Center. A colony of Germ-free (GF) B6 mice was established at POSTECH from breeders obtained from Dr. Andrew Macpherson. GF B6 mice were maintained in sterile flexible film isolators (Class Biological Clean Ltd., USA) and GF status was regularly monitored by culturing feces of GF mice. AF mice were generated and maintained in sterile flexible film isolator as previously described (50). All animal studies were performed in accordance with the guidelines of Institutional Animal Care and Use Committee of POSTECH.

### Mouse Diets

SPF diet (38057, Purina Lab) and GF diet (2018S, Envigo) were used as normal chow diet (NCD) for the maintenance of SPF and GF mice at the animal facility of POSTECH Biotech Center. AF diet (AFD) was self-generated as previously described (50). The custom diets were generated and supplied from Envigo: amino acid-defined diet (AAD, TD.01084), AAD with 3× vitamin A (AAD^*^, TD.160107), starch deficient-AAD^*^ (AAD^*^_StF, TD.160108), and mineral mix powder (TD.94049). AFD-based diets were self-generated as follows: 22% glucose in AFD (AFD_G220, same as general AFD), 50% glucose in AFD (AFD_G500) and 50% sucrose in AFD (AFD_S500).

### Preparation and Processing of Single Cell Suspensions From Small Intestine, MLN, and Spleen

Single cell suspension from SI was prepared and processed as previously described (50). In brief, SIs were dissected and opened longitudinally after removal of Peyer's patches. Tissues were cut into pieces and incubated in PBS buffer containing 3% fetal bovine serum (FBS), 10 mM EDTA, 20 mM HEPES, 100 U/ml penicillin, 100 mg/ml streptomycin, and 1 mM sodium pyruvate with gentle stirring at 37°C for 20 min to strip intestinal epithelial cells (IECs). SI segments were collected by strainer for isolating lamina propria lymphocytes and IECs in the media were enriched by 25–40 % Percoll density gradient centrifugation. Collected SI segments were digested with 400 Mandl units/ml collagenase D (Roche, Cat no. 11088882001) and 10 μg/ml DNase I (Sigma, 04536282001) in RPMI 1640 containing 3% fetal bovine serum (FBS), 20 mM EDTA, 20 mM HEPES, 100 U/ml penicillin, 100 mg/ml streptomycin, 1 mM sodium pyruvate and 1 mM Non-essential amino acids at 37°C for 45 min with continuous stirring. To stop the enzyme digestion, EDTA was added (10 mM final concentration) and cells were further incubated for 5 min at 37°C. Cell suspensions were enriched by 40–75% Percoll density gradient centrifugation. MLNs and spleens were harvested and minced with razor blade, followed by digesting at 37°C for 20–30 min with enzyme digestion buffer that were used for SI cell preparation. For analysis of stroma cells from SI, the enzyme digestions were repeated two more times with fresh enzymes for 20 min. The remaining procedures were same as SI cell preparation.

### ALDEFLUOR Assay and Flow Cytometry

Aldehyde dehydrogenate (ALDH) activity in individual cells was measured using ALDEFLUOR kits (StemCell Technologies), according to the manufacturer's protocol with modifications. Subsequently, aldefluor-reacted cells were stained with antibodies and analyzed by flow cytometry. CD16/32 Fc Blocker (93, Cat. No. 11302), CD3e-PerCp-Cy5.5 (145-2C11, Cat. No. 100328), Thy1.2-PerCp-Cy5.5 (53-2.1, Cat. No. 140322), B220-PerCp-Cy5.5 (RA3-6B2, Cat. No. 103236), NK1.1-PerCp-Cy5.5 (PK136, Cat. No. 108728), MHC class II-eFluor 450 (M5/114.15.2, Cat. No. 48-5321-82), CD11c-PE-Cy7 (N418, Cat. No. 25-0114-82), CD103-PE (2E7, Cat. No. 12-1031-82), CD11b-APC (M1/70, Cat. No. 17-0112-82), and CD8-BV650 (53-6.7, Cat. No. 100742).

Fluorochrome-conjugated antibodies for single-cell staining were purchased from BD Biosceineces, BioLegend, Thermo Fisher Scientific and Tonbo Biosciences. For LP-DC phenotypic analysis, cells were stained with antibodies and analyzed by flow cytometry: CD16/32 Fc Blocker, CD3e-PerCp-Cy5.5, Thy1.2-PerCp-Cy5.5, B220-PerCp-Cy5.5, NK1.1-PerCp-Cy5.5, CD11c-PE-Cy7, MHC class II-APC-eFluor 780 (M5/114.15.2, Cat. No. 47-5321-82), CD103-BV510 (2E7, Cat. No. 121423) and CD11b-FITC (M1/70, Cat. No.35-0112), Siglec-F-Alex Fluor 647 (E50-2440, Cat. No. 562680), Ly6C-PB (HK1.4, Cat. No. 128014), and CX3CR1-PE (SA011F11, Cat. No. 149006). For IECs and LP-SCs analysis, cells were stained with CD45-PerCp-Cy5.5 (30-F11, Cat. No. 103132), EpCAM1-eFluor 450 (G8.8, Cat. No. 48-5791-82), Pdpn-PE (8.1.1, Cat. No. 127408), and CD31-APC (390, Cat. No. 102410). For intracellular cytokine staining, cells were stimulated with eBioscience cell stimulation cocktail plus protein transport inhibitors (Thermo Fisher Scientific) for 4 h. Stimulated cells were stained with antibodies for surface markers followed by fixation and permeabilization with manufacturer's instruction (BD Biosciences). Intracellular staining with Foxp3, Tbet, and GATA3 were performed by eBioscience Foxp3/Transcription factor staining buffer set (Thermo Fisher Scientific): CD4-APC-Cy7 (RM4-5, Cat. No. 25-0042), TCR-β-PE-Cy7 (H57-597, Cat. No. 25-5961-82), CD44-eFluor 450 (IM7, Cat. No. 48-0441-82), CD62L-FITC (MEL-14, Cat. No. 104406), Nrp-1-PE (3E12, Cat. No. 145204), Foxp3-PE-Cy5.5 (FJK-16s, Cat. No. 35-5773-82), GATA3-PE (TWAJ, Cat. No. 12-9966-42), Tbet-APC (4B10, Cat. No. 644814). Dead cells were excluded by Ghost Dye (Tonbo Biosciences) or Propidium iodide (PI, Sigma) staining. Stained cells were analyzed by LSRFortessa or FACSCantoII (BD Biosciences) and data were analyzed by FlowJo software (Tree Star).

### RNA Isolatio n and Real-Time PCR

CD11c^+^MHC-II^+^CD103^+^CD11b^+^ siLP-DCs were sorted with a Moflo XDP (Beckman Coulter) and RNA was extracted using TriZol (Invitrogen). IECs were obtained after stripping SI by EDTA and RNA was extracted using RNeasy Mini kit (Qiagen) or Hybrid-R^TM^ (GeneAll). Complementary DNA was generated using QuantiTect Reverse Transcription Kit (Qiagen) or Improm II Reverse Transcriptase (Promega). Target messenger RNA was quantified using SYBR green master mix (Applied Biosystems or Takara) and gene specific primers in a duplex reaction with Hprt (TaqMan gene expression assays, Thermo Fisher Scientific): Hprt (Mm01545399_m1), (Aldh1a1 Mm00657317_m1), Aldh1a2 (Mm00501306_m1), Ido1 (Mm00492586_m1), and Tgfb1 (Mm01178820_m1). Data were analyzed on Applied Biosystems ViiA^TM^ 7 Real-Time PCR system (Applied Biosystems) or Rotor-Gene Q (Qiagen). Changes in gene expression were calculated by the comparative C_T_ method and fold changes were calculated using 2^−ΔΔCt^ method.

### *In vitro* Experiments

Common RPMI 1640 Medium (Gibco, 11875119) contains 11 mM glucose, but RPMI 1640 Medium, no glucose (Gibco, 11879020) does not contain glucose. Retinoic acid (RA, Sigma) and Glucose (Sigma) were treated to culture cells with a various concentration. SiLP-DCs, MLN-DCs and SPL-DCs were purified using anti-CD11c magnetic beads and MACS columns (Milteny Biotec). Purified DCs and SI cell suspensions (2 × 10^5^ per well in a 96-well plate) were cultured in glucose-free media with different concentration of glucose (0, 11, 22, and 44 mM) in the presence or absence of RA (1 nM). After overnight culture (20 h), cells were analyzed for RALDH activity using ALDEFLUOR assay kit. For *in vitro* Treg differentiation assay, DCs from siLP or MLN were purified in the same way as above. Purified DCs (2 × 10^4^ per well in a 96-well plate) were cultured in glucose-free media with glucose (22 mM) in the presence or absence of RA (1 nM) for 14 h. Then, they were co-cultured with 2 × 10^5^ naïve CD4^+^ T cells in the presence of 0.1 μg/ml anti-CD3, 0.1 ng/ml TGFβ-1 and 100 U IL-2. Cultures were incubated for a total of 3 days followed by FACS analysis.

### Statistical Analyses

Mean and SEM values were calculated. Two-tailed unpaired *t*-tests and one-way or two-way ANOVA with Turkey's multiple comparison test were performed, with GraphPad Prism Software.

## Data Availability Statement

All datasets presented in this study are included in the article/Supplementary Material.

## Ethics Statement

The animal study was reviewed and approved by Institutional Animal Care and Use Committee of POSTECH.

## Author Contributions

H-JK and S-WH designed, performed experiments, and analyzed data. RV performed *in vitro* iTreg differentiation experiments and analyzed data. JJ, ML, NK, and DK performed isolation of intestinal epithelial and lamina propria cells. KK performed gene expression experiments on intestinal DCs and analyzed data. CS along with H-JK initiated the study and supervised it in the initial days. DR and S-HI supervised and finalized the study to its completion. H-JK, S-HI, and DR wrote the paper. All authors contributed to the article and approved the submitted version.

## Conflict of Interest

S-HI is the CEO of the ImmunoBiome, but declares no conflicts of interest for this paper. The remaining authors declare that the research was conducted in the absence of any commercial or financial relationships that could be construed as a potential conflict of interest.
