# Supplementary material for: Dietary Glucose Consumption Promotes RALDH Activity in Small Intestinal CD103+CD11b+ Dendritic Cells
Source: Front Immunol. 2020 Aug 11;11:1897. doi: 10.3389/fimmu.2020.01897 (PMC7433714; doi:10.3389/fimmu.2020.01897)
Supplement: Supplementary file 1 [file Data_Sheet_1.pdf]

Supplementary materials

Dietary glucose consumption promotes RALDH activity in small intestinal $CD103^+CD11b^+$ Dendritic cells

Hyun-Ja Ko[1,§,#], Sung-Wook Hong[1,¶,#], Ravi Verma[2,3], Jisun Jung[2], Minji Lee[2], Nahyun Kim[2], Daeun Kim[2], Charles D. Surh[1,2,†], Kwang Soon Kim[2], Dipayan Rudra[2,*], Sin-Hyeog Im[2,3,*]

[1]Academy of Immunology and Microbiology, Institute for Basic Science, Pohang, Republic of Korea
[2]Division of Integrative Biosciences and Biotechnology & Department of Life Sciences, Pohang University of Science and Technology, Pohang, Republic of Korea
[3]ImmunoBiome Inc. 77 Cheongam-Ro, Pohang, Republic of Korea

[§] Current affiliation: KoBioLabs Inc., Seoul 151742, Republic of Korea
[¶] Current affiliation: Department of Microbiology and Immunology, Centre for Immunology, University of Minnesota Medical School, Minneapolis, Minnesota, US
[†] Deceased
[#] These authors contributed equally in this work
[*] Correspondence:
Sin-Hyeog Im (iimsh@postech.ac.kr)
Dipayan Rudra (dipayanrudra@gmail.com, rudrad@postech.ac.kr)

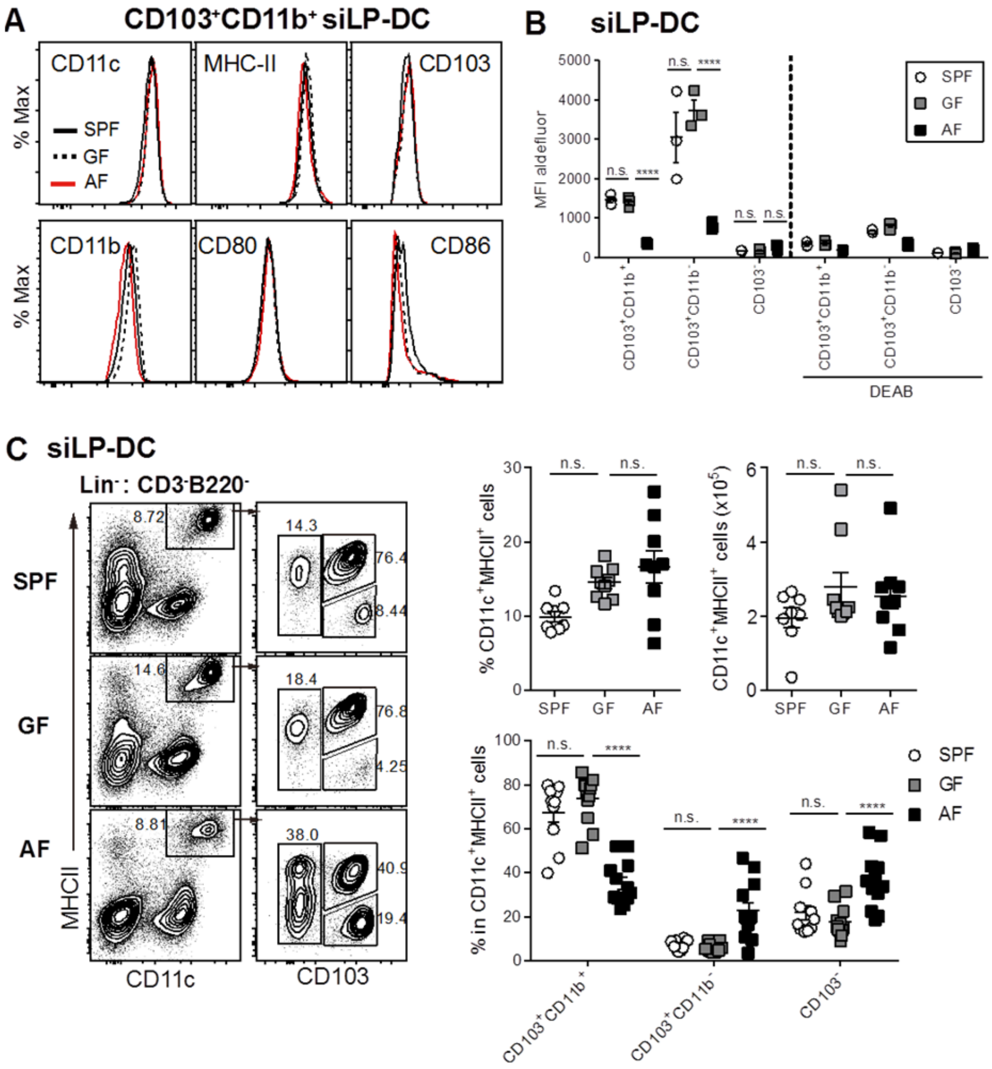

Figure S1. Altered phenotype of siLP-DC subsets in AF mice. Cell suspensions from siLP were prepared from age-matched adult (6~12-week-old) SPF, GF and AF mice, following which phenotypic and RALDH activity analyses of siLP-DCs were carried out. (**A**) Representative histograms showing expression of indicated surface markers within CD103^{+}CD11b^{+} siLP-DCs. Black lines indicate SPF, blue lines indicate GF and red lines indicate AF mice. (**B)** Statistical quantification for MFI of siLP-DC subsets. DEAB is a RALDH inhibitor. (**C**) Representative FACS plots (left) and statistical quantification (right) of siLP-DC subsets within Lin-CD11c^{+}MHC-II^{+} cells after ALDEFLUOR assay. Lin^{-} includes populations with no expression for CD3 and B220. Percentage and total numbers of CD11c^{+}MHC-II^{+} cells and siLP-DC subsets are shown. Data shown are representative of 2-3 experiments. MEAN ± SEM are indicated. Statistical significance was determined by one-way ANOVA and two-way ANOVA with Turkey's multiple comparison test. $****p < 0.0001$, *n.s.*, not statistically significant.

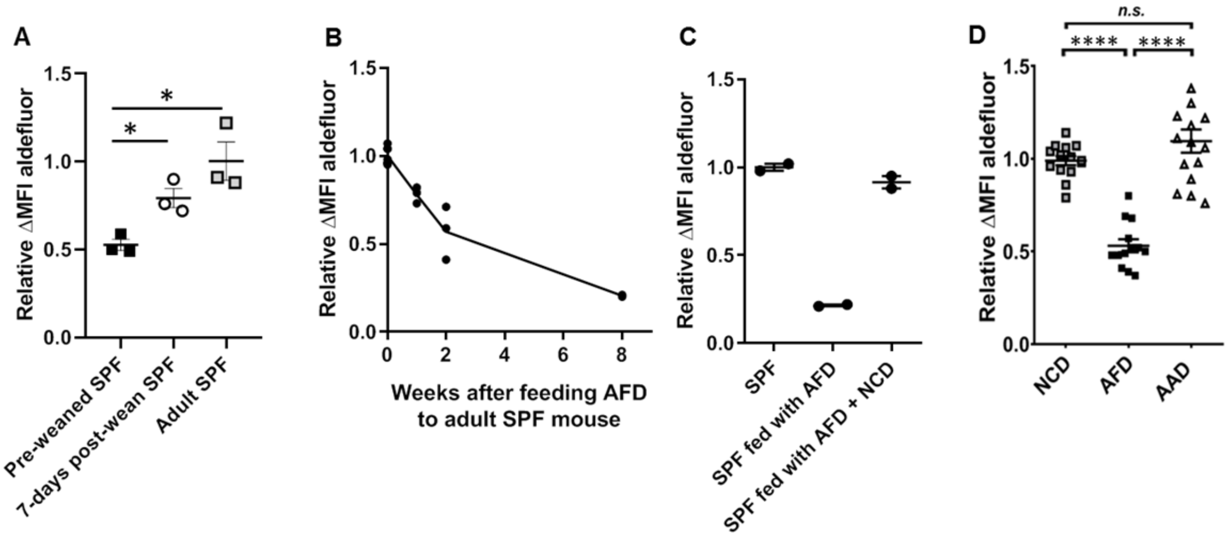

Figure S2. Dietary component mediated increase in RALDH activity in siLP-DCs in mice is independent of microbiota. Cell suspensions prepared from siLP were subjected to ALDEFLUOR assays and RALDH activity in $CD103^{+}CD11b^{+}$ siLP-DCs were analyzed by flow cytometry. (A) Pre-weaned SPF mice (3-weeks old), SPF mice weaned onto NCD for 7 days and adult SPF mice were analyzed. (B) RALDH activity in $CD103^{+}CD11b^{+}$ siLP-DCs from adult 8-12 weeks old SPF mice after feeding AFD for 0, 1, 2, and 8 weeks. (C) RALDH activity in $CD103^{+}CD11b^{+}$ siLP-DCs in adult SPF mice at the start of the experiment, after they were fed AFD for 8 weeks, and after the mice were reverted back to NCD for one week. (D) SPF mice were weaned onto AFD (22% glucose) or AAD (50% sucrose) for 4 weeks. MEAN ± SEM are indicated. Statistical significance was determined by one-way ANOVA with Turkey's multiple comparison test. *$p < 0.05$, ****$p < 0.0001$, n.s., not statistically significant.

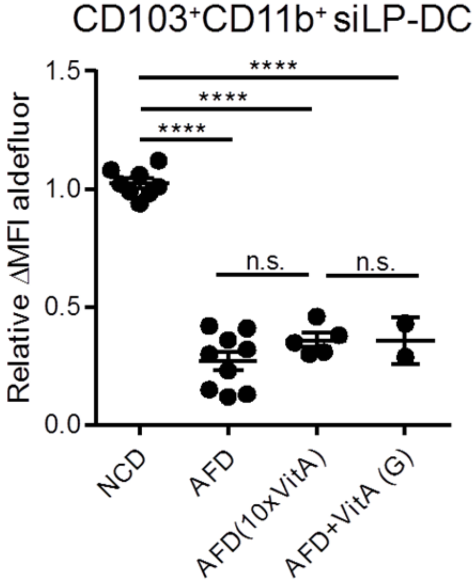

Figure S3. Supplementing AFD even with large excess of Vitamin A is unable to restore RALDH activity in $CD103^{+}CD11b^{+}$ siLP-DCs. Neonate GF mice (3-week-old) were weaned onto specific diets for 3~4 weeks. Normal AFD comprises of water soluble dietary components in water and water insoluble dietary components (including vitamin A) in soybean oil-mix. AFD(10xVitA) indicates mice fed with oil-mix containing 10 times more of vitamin A than normal AFD. AFD+VitA(G) indicates mice administrated with oil-mix (300 µl) by oral gavage for 9 days under normal AFD feeding condition. Cell suspensions from siLP were subjected to ALDEFLUOR assays and RADLH activity in $CD103^{+}CD11b^{+}$ siLP-DCs was analyzed. Data are combined from two or three independent experiments. MEAN ± SEM are indicated. Statistical significance was determined by one-way ANOVA with Turkey's multiple comparison test. $****p < 0.0001$, *n.s.*, not statistically significant.

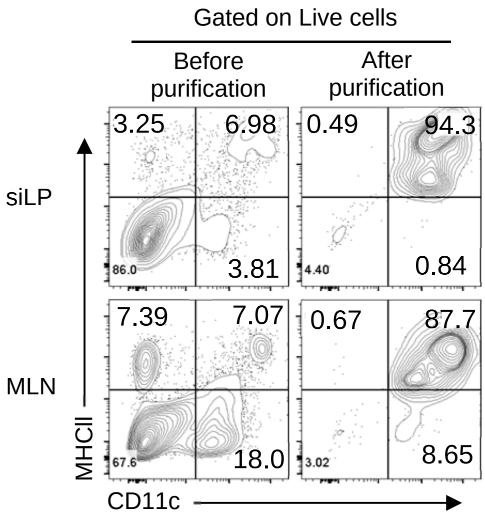

Figure S4. Purity of MLN and siLP DCs used for in vitro iTreg assay. CD11c mediated magnetic purification was performed from *wild type* C57BL/6 mice using commercially available purification kit.

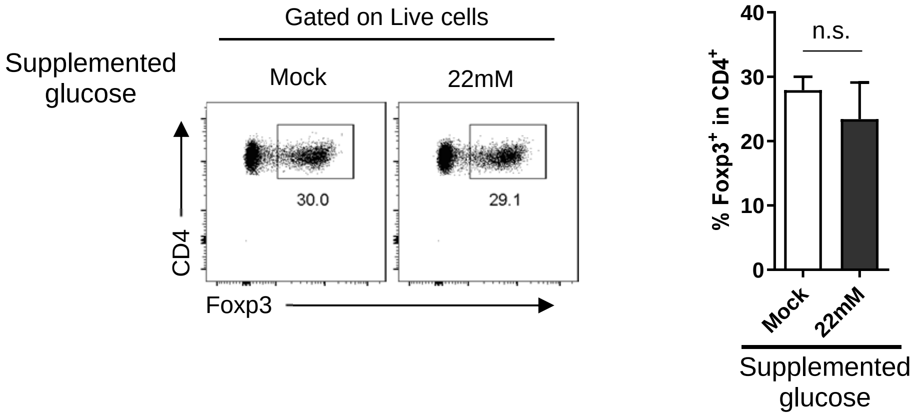

Figure S5. Glucose supplementation in a DC independent culture has little effect on in vitro iTreg induction. Sorted naïve T cells were subjected to DC independent iTreg induction with plate bound anti-CD3/ CD28, TGFβ and IL2, in the presence (22mM) or absence (mock) of excess glucose for three days. Representative FACS plots (left) and statistics (right) are shown. *n.s.*, not statistically significant.

Table S1. Vitamin A concentrations among different diet types

	SPFD	GFD	AFD
Supplier	Purina Lab. 38057	Harlan-Teklad (2018s)	AIM/IBS (Kim *et al.* 2016)
Vitamin A (IU/g)	28	30	104
Final consumption of Vitamin A per day	56 IU/2g	~ 30 IU/2g (after autoclave)	~ 31.2 IU/300 ul

Table S2. Comparison between food compositions of different modified diet types

		**NCD**<br>Harlan-Tekad<br>(2018s)	**AFD**<br>AIM/IBS<br>(Kim et al. 2016)	**AAD***<br>Harlan-Tekad<br>(TD.160107)	**AAD*_StF**<br>Harlan-Tekad<br>(TD.160108)
**Macro-nutrients**	Carbo-hydrate	Carbohydrate: 58 %<br>Protein: 24 %<br>Fat: 18 %	Glucose (22 %)	Sucrose (37 %)<br>Maltodextrin (15 %)<br>Corn starch (15 %)	Sucrose (52 %)
	Protein	Ground wheat<br>Wheat middlings<br>Ground corn<br>Corn gluten meal<br>Soybean meal<br>Brewers yeast<br>Soybean oil	**Amino acids**<br>L-Alanine<br>L-Arginine HCl<br>L-Asparagine<br>L-Glutamic Acid<br>Glycine<br>L-Histidine HCl<br>L-Isoleucine<br>L-Lysine HCl<br>L-Methionine<br>L-Phenylalanine<br>L-Proline<br>L-Serine<br>L-Threonine<br>L-Tryptophan<br>L-Tyrosine<br>L-Valine<br>L-Aspartic Acid<br>L-Cystine<br>L-Leucine	**Amino acids**<br>Alanine<br>Arginine-HCl<br>Asparagine<br>Na-Glutamate<br>Glycine<br>Histidine-HCl.H2O<br>Isoleucine<br>Lycine-HCl<br>Methionine<br>Phenylalanine<br>Proline<br>Serine<br>Threonine<br>Tryptophan<br>Ethyl L-Tyrosinate-HCl<br>Valine	
	Fat		Soybean oil	Soybean oil	
**Micro-nutrients**	Minerals	Magnesium oxide<br>Calcium iodate<br>Ferrous sulfate<br>Manganous oxide<br>Zinc oxide<br>Copper sulfate<br>Calcium carbonate<br>Dicalcium phosphate<br>Calcium carbonate<br>Iodized salt<br>Cobalt carbonate<br>Kaolin	Mg glycerophosphate<br>KI<br>Ferrous gluconate<br>Mn(acetate)H2O<br>ZnSO4H2O<br>Cu(acetate)2H2O<br>Ca glycerophosphate<br>CaCl2-H2O<br>SnSO4 2H2O<br>NaCl<br>NaF<br>(NH4)6Mo7O24 4H2O<br>Na2SeO3<br>K acetate<br>NiCl2 3H2O<br>Cr(acetate)2H2O<br>Na3VO4 (Na4VO3)<br>Co(acetate)2 4H2O	**Mineral Mix (TD.94049)**<br>Magnesium Oxide<br>Potassium Iodate<br>Ferric Citrate<br>Manganous Carbonate<br>Zinc Carbonate<br>Cupric Carbonate<br>Calcium Carbonate<br>Potassium Phosphate, monobasic<br>Potassium Sulfate<br>Sodium Chloride<br>Sodium Fluoride<br>Ammonium Paramolybdate, tetrahydrate<br>Sodium Selenate<br>Potassium Citrate, monohydrate<br>Nickel Carbonate Hydroxide, tetrahydrate<br>Chromium Potassium Sulfate, dodecahydrate<br>Ammonium Meta-Vanadate<br>Lithium Chloride<br>Boric Acid<br>Sodium Meta-Silicate, nonahydrate	
	Vitamins	vitamin B12 supplement<br>biotin<br>folic acid<br>thiamin mononitrate<br>pyridoxine hydrochloride<br>riboflavin<br>niacin<br>calcium pantothenate<br>vitamin A acetate<br>vitamin D3 supplement<br>menadione sodium bisulfite<br>vitamin E acetate<br><br>choline chloride	B12 Crystalline<br>d-Biotin<br>Folic acid<br>Thiamine HCl<br>Pyridoxine HCl<br>Riboflavin<br>Niacinamide<br>d-pantothenic acid<br>Vitamin A<br>Vitamin D3<br>Vitamin K<br>DL-Tocopherol acetate<br>DL-Tocopherol<br>Choline HCl<br>myo-Inosito	**Vitamin Mix (TD.94047)**<br>Vitamin B12<br>Biotin<br>Folic Acid<br>Thiamin HCl<br>Pyridoxine HCl<br>Riboflavin<br>Niacin<br>Calcium Pantothenate<br>Vitamin A Palmitate<br>Vitamin D3, cholecalciferol<br>Vitamin K1, phylloquinone<br>Vitamin E, DL-alpha tocopheryl acetate	
		Vitamin A: 30 IU/2g/day	Vitamin A<br>: 31.2 IU/300μl/day	Vitamin A: 23.3 IU/2g/day	
